# Reconstruction and Functional Annotation of P311 Protein–Protein Interaction Network Reveals Its New Functions

**DOI:** 10.3389/fgene.2019.00109

**Published:** 2019-02-19

**Authors:** Song Wang, Xiaorong Zhang, Fen Hao, Yan Li, Chao Sun, Rixing Zhan, Ying Wang, Weifeng He, Haisheng Li, Gaoxing Luo

**Affiliations:** ^1^Institute of Burn Research, State Key Laboratory of Trauma, Burn and Combined Injury, Southwest Hospital, Third Military Medical University, Chongqing, China; ^2^Laboratory Center of Southwest Hospital, Third Military Medical University, Chongqing, China; ^3^The Sixth Resignation Cadre Sanatorium of Shandong Province Military Region, Qingdao, China; ^4^The 324th Hospital of Chinese People’s Liberation Army, Chongqing, China

**Keywords:** P311, protein–protein interaction networks, inflammatory response, cell proliferation, coagulation

## Abstract

P311 is a highly conserved multifunctional protein. However, it does not belong to any established family of proteins, and its biological function has not been entirely determined. This study aims to reveal the unknown molecular and cellular function of P311. OCG (Overlapping Cluster Generator) is a clustering method used to partition a protein-protein network into overlapping clusters. Multifunctional proteins are at the intersection of relevant clusters. DAVID is an analytic tool used to extract biological meaning from a large protein list. Here we presented OD2 (OCG + DAVID + 2 human PPI datasets), a novel strategy to increase the likelihood to identify biological functions most pertinent to the multifunctional proteins. The principle of OD2 is that OCG prepares the protein lists from multifunctional protein relevant overlapping clusters, for a functional enrichment analysis by DAVID, and the similar functional enrichments, which occurs simultaneously when analyzing two human PPI datasets, are supposed to be the predicted functions. By applying OD2 to two reconstructed human PPI datasets, we supposed the function of the P311 in inflammatory responses, cell proliferation and coagulation, which were confirmed by the following biological experiments. Collectively, our study preliminarily found that P311 could play a role in inflammatory responses, cell proliferation and coagulation. Further studies are required to validate and elucidate the underlying mechanism.

## Introduction

P311, with the official gene symbol NERP (neuronal regeneration related protein), is a highly conserved 8-kDa intracellular protein. The 68-amino acid sequence of P311 contains a PEST domain (rich in Pro, Glu, Ser, and Thr) in the N-terminus (Stradiot et al., 2018). The domain is also in short-lived proteins such as transcription factors, cytokines and signal molecules, which implies that P311 might belong to one of the protein families (Sommer and Wolf, 2014; Varshavsky, 2014). However, no more evidence was found to ascribe P311 to one of the protein families. So far, P311 does not belong to any established family of proteins; therefore, it fails to provide any clues on its function. Studler et al. (1993) first reported that P311 was highly expressed in embryonic mouse brains, and then other groups demonstrated that P311 was expressed in motoneurons (Fujitani et al., 2004), glioblastomas (McDonough et al., 2005), smooth muscle cells (Badri et al., 2013), and fibroblasts (Tan et al., 2010; Cheng et al., 2017). Furthermore, these studies showed that P311 was involved in nerve and lung regeneration (Fujitani et al., 2004; Zhao et al., 2006), glioma invasion (Mariani et al., 2001; McDonough et al., 2005), blood pressure homeostasis (Badri et al., 2013), myofibroblast differentiation (Pan et al., 2002), amoeboid-like migration (Shi et al., 2006), behavioral responses in learning and memory (Taylor et al., 2008) and the affective, but not the sensory component of pain (Sun et al., 2008).

Our group has been focused on P311 since 2004, when we found that the expression of P311 increased dramatically in hypertrophic scars through gene expression profiling and a comparative proteomics analysis (Wu et al., 2004). We then found that P311 induced fibroblast differentiation via enhancing the TGFβ1 signaling pathway in a human hypertrophic scar (Tan et al., 2010), and it also was a new inducer of EpMyT (Epidermal stem cell transdifferentiate into myofibroblasts) in wound healing (Li et al., 2016). Furthermore, we demonstrated that P311 played a crucial role in renal fibrosis via TGFβ1/Smad signaling (Yao et al., 2015). Recently we showed that P311 accelerated skin wound re-epithelialization by promoting epidermal stem cell migration through RhoA and Rac1 activation (Yao et al., 2017) and that P311 deficiency leads to attenuated angiogenesis in cutaneous wound healing (Wang et al., 2017). These studies have aroused our tremendous and sustained interest in P311 and we therefore continue to study its biological function.

Protein-protein interaction (PPI) networks can highlight the modularity of cellular processes and allow the deciphering of protein functions at the cellular level, as proteins tend to interact with each other when they are involved in the same molecular complex, pathway, or biological process (Hartwell et al., 1999). Meanwhile, a PPI network can be represented as a simple graph in which vertices correspond to proteins and edges, to direct physical interactions, which allow the graph partition method to highlight clusters of densely connected vertices (Aittokallio and Schwikowski, 2006). The identified clusters stand for groups of proteins involved in the same molecular complex, pathway, or biological process. Further analyzing the proteins from each group can predict the function of uncharacterized proteins (Sharan et al., 2007). OCG (Overlapping Cluster Generator) is a graph partition that decomposes a protein-protein network into overlapping clusters. Multifunctional proteins are at the intersection of relevant clusters (Becker et al., 2012). DAVID consists of a comprehensive biological knowledgebase as well as analytical tools designed to systematically extract biological meaning from a large gene/protein list (Huang et al., 2008).

In this study, we presented OD2 (OCG + DAVID + 2 human PPI datasets), a promising strategy to predict the function of P311. By applying OD2 to two reconstructed human PPI datasets, we supposed the function of P311 in inflammatory responses, cell proliferation and coagulation. Finally, we conducted relevant biological experiments to confirm the functions of P311.

## Materials and Methods

### Datasets

**FIGURE 1:**
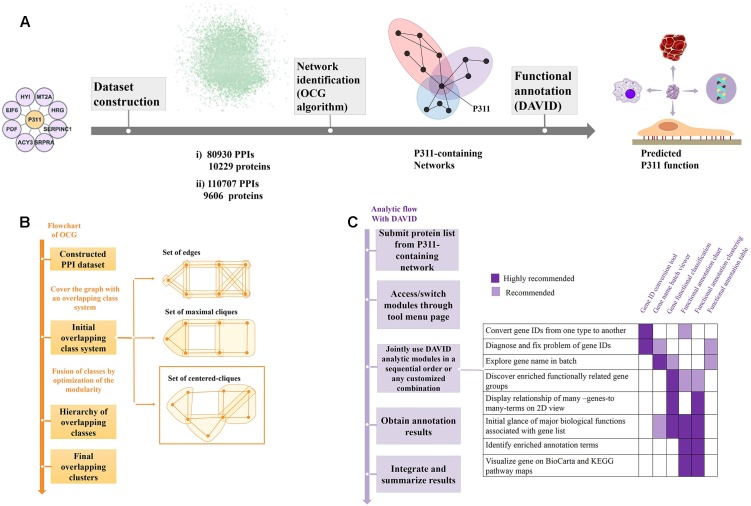
Flow diagram of the bioinformatic analysis. **(A)** Flow diagram of OD2 approach. The initial P311-containing network identified in the Y2H screen (Peng et al., 2012) were integrated with the human PPI dataset gathered by Bossi and Lehner (2009) and dataset from the STRING database (Szklarczyk et al., 2014), separately. The resulting datasets were decomposed and identified the reconstructed P311-containing network using an OCG algorithm. The proteins in each reconstructed P311-containing network were analyzed with DAVID. Finally, predicted functions of P311 were identified. **(B)** Flowchart of the OCG (Overlapping Cluster Generator) algorithm. A graph was covered by the initial overlapping class system with centered cliques. Based on optimization of modularity of the partition, a hierarchy of nested clusters was built iteratively, leading to the final overlapping clusters (Becker et al., 2012). **(C)** Analytic flow with DAVID (Huang et al., 2008).

A PPI network dataset (named Dataset 1) involving 80,930 binary interactions between 10,229 proteins (Figure 1A and Supplementary Material 1) was constructed by (1) eight interactions from our previous literature (Peng et al., 2012) and (2) 80,922 binary interactions from the PPI network dataset assembled by Bossi and Lehner (2009).

Additionally, another high confidence dataset (named Dataset 2) of 110,707 binary interactions involving 9,606 proteins (Figure 1A and Supplementary Material 3) was built by fusing the eight interactions from our previous literature (Peng et al., 2012) with 110699 binary interactions, whose combine-score is greater or equal to 0.5, from the STRING database (Szklarczyk et al., 2014). In the STRING dataset, each protein-protein interaction is annotated with a combine-score. The score does not necessarily indicate the strength or specificity of the interaction, instead, it indicates confidence, i.e., how likely STRING judges an interaction to be true, given the available evidence. All scores rank from 0 to 1, with 1 being the highest possible confidence.

### OCG (Overlapping Cluster Generator) Algorithm

The OCG algorithm was carried out by the software available in Becker et al. (2012) (Supplementary Material 4). The principle of OCG is to build a tree in which the leaves are introductory classes that are progressively and hierarchically joined (Figure 1B).

#### Initial Overlapping Class System

This program begins with building initial overlapping clusters from a simple unweighted graph *G* = (*V*, *E*). Let |*V*| = *n* and |*E*| = *m*. To obtain the initial overlapping class system, there are three strategies to cover the graph.

The covering class system can be:

(1) Set of edges: The Newman’s algorithm (Newman, 2006) continues to search for two clusters *V_i_* and *V_j_* such that ∀ (*x,y*) ∈ *V_i_* × *V_j_*, (*x,y*) ∈ *E* to maximizes the modularity *Q* of the system.
(2) Set of maximal cliques: When computing the maximal cliques, a class system with maximal modularity *Q* is formed. Any class fusion will cause *Q* to decrease until $Q_{\min}=\Sigma_{x=1-n}d_{x}^{2}$.
(3) Set of centered cliques: For each vertex x ∈ G, a greedy polynomial algorithm is used to construct a clique. As long as a clique is generated, the vertices adjacent to *x* are arranged in descending order of the relative degree. The resulting clique that contains *x* is not necessarily the maximal one, as there may be a larger one containing *x*.

The centered clique system has been chosen for further studies, because its graph density is better and is less time and memory consuming.

#### Hierarchy of Overlapping Class

To obtain the hierarchy overlapping class systems, the need to be fused with the clusters by optimization of the modularity *Q*. In each step, the connected clusters are the ones that maximize the average gain. This average is defined as the global modular gain divided by the number of newly connected vertex pairs. The chain effect can thus be avoided, which is caused by adding elements one by one and producing clusters that are not suitable for the following functional prediction.

#### Final Overlapping Clusters

Final overlapping clusters come out when the system of clusters reaches the maximized modularity. Alternatively, if the researcher sets either a minimum number of clusters or the maximum allowed cluster cardinality, the final overlapping cluster that maximizes the overall modularity within those constraints come out.

### P311 PPI Networks Reconstruction and Analysis

As shown in Figure 1A, through the OCG (Overlapping Cluster Generator) algorithm, the dataset was partitioned into overlapping PPI networks (Supplementary Material 5). Among those, we picked out the networks containing protein P311 (NERP) as P311-containing networks.

Following the analysis flow with DAVID (Figure 1C), all constituents in each P311-containing network were analyzed, separately, to find the significant terms. The *Q*-values < 0.05 were invoked as the threshold from which to choose the significant terms (Katsogiannou et al., 2014). *Q*-values are hypergeometric *p*-values corrected for multiple testing according to the Benjamini and Hochberg procedure. Cytoscape was utilized to visualize all the networks (Smoot et al., 2010). Significant GO terms occurring in both terms from dataset 1 derived P311-containing networks and dataset 2 derived P311-containing networks, were picked out as the common terms, which were supposed to be the functions of P311.

Following the bioinformatic analysis above, we identified the common predicted function as the function which would be confirmed in the follow-up experiments.

### Animals

P311 WT and P311 KO mice were kindly gifted by Professor Gregory A Taylor (Taylor et al., 2008). All mice grew up in the animal Institute of Third Military (Army) Medical University. The mice were maintained in a specific, pathogen-free environment under controlled light, temperature, and humidity.

### Culture of Mouse Primary Fibroblasts

Cells were cultured as previously described (Varani et al., 2004). Briefly, after incubation in 0.25% Dispase II (04942078001. Roche) overnight at 4°C, the dermis was separated and minced into tissue fragments. Then primary fibroblasts were grown from the fragments and cultured in Dulbecco’s modified Eagle’s medium (DMEM) (11965118, Gibco) supplemented with 10% fetal bovine serum (FBS) (10099141, Gibco), 100 U/mL of penicillin and streptomycin (15140122, Gibco).

### Proliferation Assay

Mouse primary fibroblasts (MPFs) were seeded in three replicates in 96-well plates in DMEM supplemented with 10% FBS. After 1, 2, 3, 4, 5, and 6 days, according to the manufacturer, the absorbance was measured at 450nm using an enzyme-linked immunosorbent assay reader with the addition of 10 μl/well of CCK8 reagent (CK04, Dojindo).

### Full-Thickness Excisional Skin Wound Model

The model was prepared as previously described (Wang et al., 2018). Briefly, hairs on the dorsal surface of mice were shaved with an electric shaver and cleaned with 75% alcohol before surgery. A full-thickness excisions skin wound was made on the dorsal surface, with a 4 mm round skin biopsy punch, while anesthetized, with 1% pentobarbital via an intraperitoneal injection.

### Superficial Second-Degree Burn Mouse Model

As previously described (Li et al., 2016), mice were anesthetized with intraperitoneal pentobarbital (35 mg/kg) and dorsal skin hairs were shaved 2 days before the burn. The scald apparatus (YSL-5Q) was then used to make the second-degree thermal burn injury with the condition (80°C, 3 s under a pressure of 500 g weight). Two wounds were produced on each mouse along the posterior median line, and the distance between the two wounds was 1.0 cm. The burn depth was confirmed by pathology.

### Hematoxylin-Eosin (H&E) Staining

The mice were sacrificed on the 3rd-day post-surgery, and the wound tissues were then carefully harvested, fixed with 4% paraformaldehyde, embedded in paraffin, sliced and stained with H&E. The wound area and inflammatory cell count on the hematoxylin and eosin (H&E) stained sections were determined by the ImageJ 1.41 software provided by the National Institute of Health.

### RNA Isolation and Quantitative Real-Time PCR

Total RNA was extracted from mouse skin with the RNeasy Mini Kit (QIAGEN, 74104), and cDNA was synthesized with the cDNA Synthesis Kit (Toyobo, FSK-100). Real-time PCR was performed with the SYBR Green Master Mix (Toyobo, QPK-201) on a 7500 Real-Time PCR System (Applied Biosystems Instruments). The following primers were used:

CD14, 5′-ACATCTTGAACCTCCGCAACGTGT-3′ and 5′TTGAGCGAGTGTGCTTGGGCAATA-3′; CD16, 5′-TTGCAGTGGACACGGGCCTTTATT-3′ and 5′TTGTCTTGAGGAGCCTGGTGCTTT-3′; β-actin, 5′-CGTGCGTGACATCAAAGAGAA-3′ and 5′-TGGATGCCACAGGATTCCCAT-3′; GAPDH, 5′-CGTGCCGCCTGGAGAAAC-3′ and 5′-AGTGGGAGTTGCTGTTGAAGTC-3′; P311, 5′-GAGGCTTCCTAAGGGAAGACTT-3′ and 5′-AAGTGGAGGTAAC TGATTCTTGG-3′.

### Flow Cytometry

After isolating cells from the dermal sheet, the appropriate primary antibody was added for 1 h at 4°C to PerCP CY5.5-conjugated mAb specific F4/80 (Cell Signaling). After transduction with Ad-P311 or Ad-Vector for 24 h, MPFs (mouse primary fibroblasts) were scraped off the plates in PBS containing 5% BSA. Cells were then fixed in 70% ethanol in 4°C overnight, washed in PBS two additional times, and then stained for 30 min at 37°C in a 50 mg/ml propidium iodide (Sigma, United States) solution containing 200 mg/ml RNase A and 0.1% Triton-X-100. All the prepared cells were analyzed with the Attune Acoustic Focusing Cytometer (Applied Biosystems, Life Technologies, CA, United States), and the data were analyzed using the Flow Jo software (Tree Star Incorporation, United States). Experiments were replicated at least three times using the same conditions and settings.

### Thromboelastography (TEG)

Thromboelastography (TEG) is a method of testing the efficiency of blood coagulation (Branco et al., 2014). Blood was gathered from the retro-orbital plexus of the P311 WT-burn, P311 KO-burn, P311 WT-sham-burn, and the P311 KO-sham-burn mice on the 7th-day post-burn, in tubes containing buffered sodium citrate. A minimum of 1 mL was achieved in each case. The previously described (Bolliger et al., 2012), the TEG analysis at 37°C using the ‘Citrated Native’ protocol with TEG 5000 Thromboelastography Hemostasis Analyzer System (Haemonetics Corporation, Braintree, MA, United States) was used. Descriptions of the TEG parameters are provided in Table 1.

**Table 1:** Description of thromboelastography (TEG) parameters.

Parameter	Description
Reaction time (R, min)	The speed of initial clot formation
Angle (α- Angle, degrees)	A measure of clot rate, the tangent of the curve at 2 mm amplitude
Maximum amplitude (MA, mm)	A reflection of clot strength, the maximum clot strength
*G*-value (*G*,*k*)	A measure of clot strength, calculated overall clot strength
Per cent lysis at 30 min (LY30, %)	A measure of blood clot dissolution, the measure of per cent clot lysis 30 min after MA
Per cent lysis at 60 min (LY60, %)	A measure of blood clot dissolution, the measure of per cent clot lysis 60 min after MA


### Statistical Analysis

For DAVID analysis, the entire genome-wide genes of humans were the default background, and the significance of the gene-term enrichment was analyzed by a modified Fisher’s exact test (EASE score). For follow-up experiments, the data were described as mean ± SD (standard deviation) and analyzed by an unpaired, two-tailed Student’s *T*-test with SPSS 18.0 software. *P* < 0.05 was considered as statistically significant.

## Results

### Functional Enrichment Analysis of P311 PPI Networks Predicts Its New Functions

Previously, our group identified eight proteins that might interact with P311, utilizing the yeast two-hybrid (Y2H) technique. These proteins are HRG, SERPINC, MT2A, SRPR, HYI, ACY3, EIF6, and PDF (Supplementary Material 2) (Peng et al., 2012). The nine proteins then constructed the initial P311-containing network. Following the analytic flow of DAVID (Figure 1C), according to functional annotation and enrichment, the proteins, with a *q*-value more than 0.05, were enriched in the negative regulation of endopeptidase activity (*Q* = 0.9404).

As shown in Figure 1A, the initial P311-containing network was merged with the human PPI network dataset assembled by Bossi and Lehner (2009) and the human PPI network dataset downloaded from STRING database (Szklarczyk et al., 2014), was used separately, to build two datasets. We obtained two reconstructed human PPI datasets, one (named Dataset 1) with 80,930 binary interactions between 10,229 proteins (Figure 1A and Supplementary Material 1), another (named Dataset 2) with 110,707 binary interactions involving 9,606 proteins (Figure 1A and Supplementary Material 3).

The two large human PPI networks were partitioned by OCG using the centered clique system to initially cover the graph. The final overlapping clusters emerged when the maximal modularity was reached (Figure 1B). Finally, 732 overlapping clusters, four of which contained P311, were obtained from Dataset 1. The four reconstructed P311-containing networks were named M1, M2, M3, M4 (Supplementary Materials 5, 6). Meanwhile, we obtained four reconstructed P311-containing networks, among 1588 overlapping clusters, obtained from Dataset 2. The four reconstructed P311-containing networks were named N1, N2, N3, N4 (Supplementary Materials 5, 7). Overall, we obtained eight reconstructed P311-containing networks.

**FIGURE 2:**
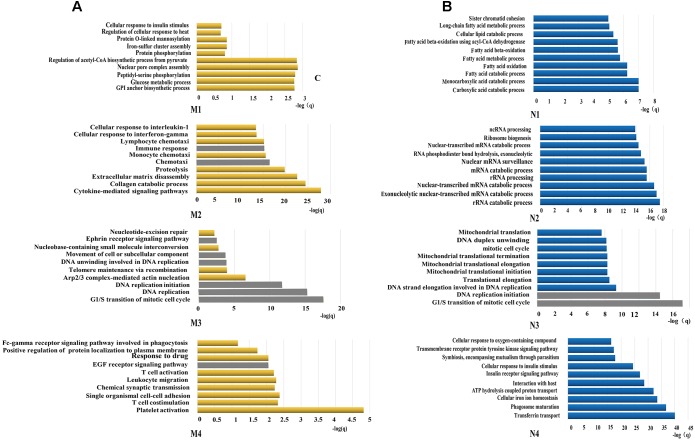
Functions predicted by the bioinformatic analysis. **(A)** Represents the top 10 predicted functions of P311 in modules from dataset 1. **(B)** Represent the top 10 predicted functions of P311 in modules from dataset 2. Gray squares stand for the predicted common functions.

All constituents in each reconstructed P311-containing network were then analyzed by DAVID, separately (Figure 1C). In the analysis of dataset 1 derived P311-containing networks, according to functional annotation and enrichment, these functions range from biological processes already reported (Supplementary Material 8) to novel ones (Supplementary Material 9), such as the GPI anchor biosynthetic process, glucose metabolic process, peptidyl-serine phosphorylation, chemokine-mediated signaling pathway, monocyte chemotaxis, cellular response to interferon-gamma, G1/S transition of mitotic cell cycle, DNA replication, platelet activation and the positive regulation of the establishment of protein localization to the plasma membrane. The top ten functions of each reconstructed P311-containing network are shown in Figure 2A.

In the analysis of dataset 2 derived P311-containing networks, according to functional annotation and enrichment, the predicted functions also included the already reported ones (Supplementary Material 8) and the new ones (Supplementary Material 10). The novel functions were enriched in the carboxylic acid catabolic process, monocarboxylic acid catabolic process, rRNA catabolic process, rRNA processing, the G1/S transition of mitotic cell cycle, DNA replication initiation and so on. The top 10 functions of each reconstructed P311-containing network are shown in Figure 2B.

To improve the confidence of the bioinformatic analysis, we compared the predicted functions from dataset 1 and dataset 2 to find the ones occurring on both sides. As shown in Table 2, one identified function (positive regulation of cell migration) (McDonough et al., 2005; Yao et al., 2017) was predicted by the analysis system. Another 13 unidentified ones were reported.

**Table 2:** Predicted function of P311.

New predicted functions of P311	Known functions of P311
• The G1/S transition of the mitotic cell cycle (M3, N3).	• Positive regulation of cell migration (M2, N2)
• Regulation of transcription involved in the G1/S transition of mitotic cell cycle 90 (M3, N3).	
• DNA replication (M3, N3).	
• DNA unwinding involved in DNA replication (M3, N3).	
• DNA replication initiation (M3, N3).	
• Movement of a cell or subcellular component (M3, N2).	
• Chemotaxis (M2, N2).	
• Immune response (M2, N2).	
• Epidermal growth factor receptor signaling pathway (M4, N2).	
• Blood coagulation (M2, N2).	
• Cellular protein metabolic process (M2, N2).	
• Innate immune response (M2, N2).	
• Ephrin receptor signaling pathway (M3, N2).	




*M, N stand for the P311-containing network from dataset one and dataset two, respectively.*

Integrated with the predicted functions above, and the phenomenon we observed before during our experiments, we supposed the function of P311 in inflammatory responses, cell proliferation and coagulation as the most confident ones.

### The Contribution of P311 to the Inflammatory Responses During Wound Healing

**FIGURE 3:**
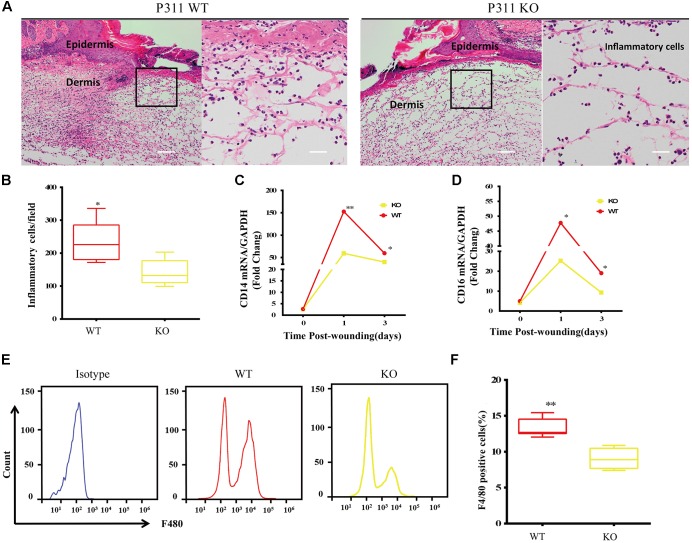
Effect of P311 on the inflammatory response during wound healing. **(A)** Representative histological analysis of a skin wound by H&E staining on the 3rd day. The area marked by the black box in the left column is enlarged to display the mononuclear inflammatory cells in the right column. Scar bar = 200 μm, 50 μm. **(B)** Quantitation of mononuclear inflammatory cells in the granulation tissues from H&E-stained wound sections on the 3rd day (*n* = 3 animals per genotype). CD14 **(C)** and CD16 **(D)** mRNA expression in wounds on days 0, 1, and 3 after injury (*n* = 3 animals per time point and genotype). GAPDH and β-actin are the housekeeping genes used to perform the qPCR analysis. **(E)** Representative flow cytometry of F4/80. **(F)** Quantitation data of flow cytometry (*n* = 3 animals per genotype). Data represented mean ± SD, Student *T*-test, ^∗^*P* < 0.05, ^∗∗^*P* < 0.01, P311 KO vs. WT.

Inflammatory responses are the hallmark pathophysiological procedure for wound healing. After injuries such as trauma, burns or surgery, the prompt recruitment of inflammatory cells, such as monocytes and macrophages, which express CD14 and CD16 markers, occur in the wound site, followed by neutrophils and lymphocytes. These cells control the inflammatory response to wounds (Eming et al., 2007). Histological analysis of the wounds was carried out to check the microscopic appearances of wounds and the number of inflammatory cells that have infiltrated into the subcutaneous areas. The results showed that on the 3rd-day after the injury, the number of inflammatory cells in P311 WT was significantly higher than that in P311 KO mice (231.5 ± 63.9/field vs. 141.4 ± 39.2/field, *p* < 0.05) (Figure 3A,B). Further, we detected the mRNA levels of CD14 and CD16 expressed in wounds from the two groups on the 1st- and 3rd- day after the injury. We found more than a 150-fold change in CD14 expression and more than a 50-fold change of CD16 expression in wounds in P311 WT mice compared with non-wounded skin (*p* < 0.05) (Figure 3C,D). Additionally, changes in P311 KO mice were considerably smaller than those in P311 WT mice. On the 3rd day after the injury, the levels of CD14 and CD16 decreased compared with those on the 1st day after injury, but the expression levels in P311 WT mice were still significantly higher than that in P311 KO mice (*p* < 0.05) (Figure 3C,D). Detail CT values are provided in Supplementary Material 11. Finally, we utilized flow cytometry to detect the percentage of F4/80+ inflammatory cells in the wounds. Consistent with the findings above, we found that wounds from P311 KO mice showed a significant reduction in the percentage of F4/80+ inflammatory cells in the wounds (13.3 ± 1.3% vs. 9.0 ± 1.4%, *p* < 0.05) on the 3rd day after injury (Figure 3E,F). All these findings confirmed that P311 influences the inflammatory responses during wound healing, by affecting the initial inflammatory cell recruitment.

### P311 Overexpression Promotes Proliferation of Mouse Primary Fibroblasts (MPF)

**FIGURE 4:**
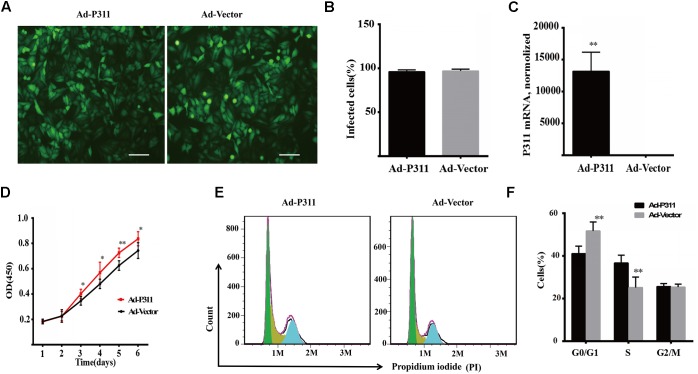
P311 overexpression promotes proliferation of primary mouse fibroblasts (MPF). **(A)** Representative morphology of primary mouse fibroblasts (MPF) transfected with Ad-P311 or a negative vector (GFP). Transfected for 48 h and then observed under a fluorescence microscope to confirm the infection efficiency by visualizing GFP expression. Scale bar = 100 mm. **(B)** Quantitation data of GFP^+^ cells fraction in P311 transfected MPFs and vector group to determine the transfection efficiency. **(C)** P311 mRNA level in P311 transfected MPFs and vector group (*n* = 4 per group). **(D)** Representative flow cytometry cell cycle. **(E)** Quantitation data of flow cytometry cell cycle (*n* = 6). **(F)** The CCK8 assay was performed to assess the effect of P311 on the proliferation (*n* = 6). ^∗^*P* < 0.05, ^∗∗^*P* < 0.01, Ad-P311 vs. Ad-Vector.

To confirm the function of P311 in proliferation, P311 was cloned into a pAdEasy vector which expresses GFP (green fluorescent protein), and then MPFs were transfected with a recombinant adenovirus vector (P311) or a negative vector (GFP). Before flow cytometry analysis and proliferation assay, the transfection efficiency was quantified and verified by observing GFP expression using a fluorescent microscope, and P311 mRNA expression levels were checked in real-time PCR. After transfection for 48 h, we observed that more than 90% of MPFs were transfected in both groups (Figure 4A,B). Real-time PCR result displayed that the mRNA level of P311 in the recombinant adenovirus vectors (P311) group was more than 10000-fold higher than that in the negative vector (GFP) group (*p* < 0.05), which confirmed the over-expression of P311 in the recombinant adenovirus vectors (P311) group. Detailed CT values are appended in the Supplementary Material 11. Further, proliferation capacity of these transfected cells was assessed using a proliferation assay. The result showed that from day 3, cells in the recombinant adenovirus vectors (P311) group had a higher proliferation capacity than cells in the negative vector (GFP) group (*p* < 0.05), and this tendency continued to the end (*p* < 0.05), which indicated that P311 over-expression significantly promoted the proliferation of MPFs (Figure 4D). To understand how P311 promoted the proliferation of MPFs, we determined the cell cycle status of transfected cells by PI (Propidium iodide) staining. We found that the negative vector (GFP) group had a higher proportion of cells in the G0/G1 phrase (51.71 ± 4.21% vs. 41.05 ± 3.49%, *p* < 0.05), while the proportion of cells in the S phase was significantly lower in the negative vector (GFP) group than in the recombinant adenovirus vectors (P311) group (25.16 ± 4.92% vs. 36.68 ± 3.56%, *p* < 0.05) (Figure 4F). Moreover, there was no difference in the proportion of cells in the G2/M between the two groups (*p* > 0.05). These finding were consistent with the prediction by bioinformatics analysis. All of which indicated that P311 promoted MPFs proliferation via enhancing cells intothe S phase.

### The Coagulation Profile of Blood Obtained From P311 WT Versus P311 KO Mice Immediately and 7-Days Post Burned

To assess the function of P311 in coagulation, we established a superficial second degree burn mouse model, as burn injury is traditionally thought to be a common triggering cause of coagulopathy, ranging from activation of coagulation to disseminated intravascular coagulation (DIC) (Curreri et al., 1975). Thromboelastography (TEG) was then used to monitor the coagulation profile of blood after trauma, as growing evidence shows that TEG is better than the conventional laboratory tests in evaluating the coagulation profile, platelet dysfunction, and fibrinolysis after trauma (Branco et al., 2014; Pekelharing et al., 2014).

**FIGURE 5:**
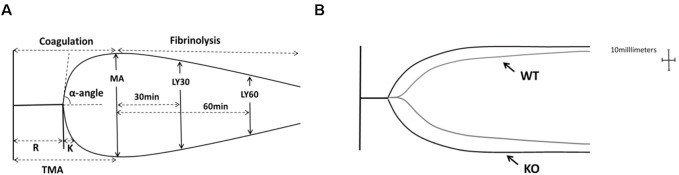
The impact of P311 knock-out on murine blood coagulation. **(A)** A schematic TEG tracing. The parameters are described in Table 1. **(B)** Quantitation of the neo-epidermal length (*n* = 6). Representative TEG tracing from a P311 WT-burn mouse (gray line) and P311 KO-burn mouse (black line). The details of the result of TGE analysis is showed in Table 2.

Figure 5A represented a schematic TEG tracing. Results of TEG analysis are shown in Table 3, while Figure 5B represents TEG tracings from the two groups. On the 7th-day post-injury, no difference was detected between the P311 WT-sham and the P311 KO-sham injury. The clot formation time (R 8.850 ± 1.115 vs. 8.167 ± 1.291 min, *p* = 0.955) was similar, and the clot rate (α- angle 6.075 ± 1.656° vs. 6.275 ± 1.819°, *P* = 0.876) was also almost the same. No fibrinolysis was observed for both the P311 WT or the P311 KO mice. Comparing the P311 WT-burn to the P311 KO-burn, except for the clot formation time (R 8.850 ± 1.541 vs. 7.733 ± 1.210 min, *p* = 0.57), other parameters (Angle, MA, and G) were all significantly different (*p* < 0.05), which implied that P311 might regulate the process of coagulation. Altogether, the experiment demonstrated that P311 knockout significantly impacts burn-induced coagulopathy, suggesting a potential target for therapy.

**Table 3:** Comparison of P311 WT and P311-KO mice after burned and sham injury.

	WT-sham (*n* = 6)	KO-sham (*n-*6)	*p*	WT-burned (*n* = 6)	KO-burned (*n* = 6)	*p*
*R* (min)	8.850 ± 1.115	8.167 ± 1.291	0.955	8.850 ± 1.541	7.733 ± 1.210	0.57
Angle, a (degrees)	6.075 ± 1.656	6.275 ± 1.819	0.876	9.075 ± 3.143	19.833 ± 2.079	0.002
MA (mm)	16.775 ± 3.132	17.100 ± 3.110	0.888	18.525 ± 3.648	34.200 ± 7.233	0.049
*G*(*k*)	377.750 ± 155.663	405.650 ± 135.393	0.796	1197.875 ± 742.662	3170.033 ± 582.418	0.005
LY30 (%)	0 ± 0	0 ± 0	NS	0 ± 0	0 ± 0	NS
LY60 (%)	0 ± 0	0 ± 0	NS	0 ± 0	0 ± 0	NS


## Discussion

In this study, bioinformatic and experimental approaches were combined to predict and validate the function of P311. Firstly, by applying OD2 to two reconstructed human PPI datasets, we predicted the function of P311 in inflammatory responses, cell proliferation and coagulation. The principle of OD2 is that OCG prepares the protein lists from multifunctional protein relevant overlapping clusters, for functional enrichment analysis by DAVID. Similar functional enrichments, which occur simultaneously when analyzing two human PPI datasets, were chosen to be the predicted functions. Finally, we conducted relevant biological experiments to confirm these functions of P311.

### Integrating Initial P311 PPI Network With the Human Interactome Strengthens the Functional Landscape of P311

As proteins tend to interact with each other when they are involved in the same molecular complex, pathway, or biological process, the understanding of protein function is intrinsically tied to the understanding of this network (Hao et al., 2016). These networks are functional units of protein–protein interaction (PPI) networks and allow function prediction when involving unidentified proteins (Brun et al., 2003; Sharan et al., 2007). DAVID consists of a comprehensive biological knowledgebase and analytical tools designed to systematically extract biological meaning from a large gene/protein list (Huang et al., 2008). To decipher the unknown function of P311, we performed functional annotation and enrichment analysis of constituent proteins in the initial P311-containing network, using DAVID. The binary analysis result showed that P311 might have the function to regulate endopeptidase activity, which was not observed in our follow-up experiments. This implied that the strategy to analyze the PPI network, which consists of binary interactions, has its own limitations, as it did not include the extended functional information hiding in the whole human PPI network, which is modular and consists of groups of highly related proteins in the same cellular function (Hartwell et al., 1999; Brun et al., 2003). Katsogiannou et al. (2014) reported that merging the initial PPI network with human interactome can enhance the functional information. Following their strategy, we merged the initial P311-containing network identified by our group, with two existing human PPI datasets, respectively (Figure 1).

Regarding a PPI network as a simple graph, in which vertices correspond to proteins and edges to direct physical interactions, allows the graph partition method to identify the networks (Newman, 2006). OCG is a clustering method used to identify the networks containing proteins involved in the same molecular complex, pathway, or biological process, and multifunctional proteins were identified at the intersection of the overlapping networks. Moreover, OCG had a better trade-off between sensitivity and specificity than the CFfinder and Link communities did (Becker et al., 2012). By applying OD (OCG + DAVID) to PPI networks, we found that the result of analyzing different datasets by OD may be a little different, as the datasets were built at a different time by different researchers, which may cause them to not all have the same data in the dataset. Reversely, this may demonstrate the reliability of the result. To improve the confidence of the bioinformatic analysis, we chose the common predicted functions occurring on both sides to be the predicted function. So the bioinformatic system was developed into OD2 (OCG + DAVID + 2 human PPI datasets) with the principle that OCG prepared the protein lists from multifunctional protein relevant overlapping clusters for functional enrichment analysis by DAVID. The similar functional enrichments, which occurred simultaneously when analyzing two human PPI datasets, were chosen to be the predicted functions (Figure 1).

Through OD2, we predicted that P311 was involved in one well-known function-positive regulation of cell migration, which supported a reliable prediction function of the system. It has been reported that P311 accelerated cell migration mainly by enhancing the activity of GTPase. Mechanisms, involving P311 in enhancing the activity of GTPase, were cell specific. In epidermal stem cells, it enhanced the activity of Rho A and Rac1 (Yao et al., 2017). While in myofibroblasts, it enhanced the activity of Ral A (Shi et al., 2006). Other known functions of P311 like the regulation of blood pressure homeostasis (Badri et al., 2013), the regulation of development of fibrosis (Yao et al., 2015; Cheng et al., 2017), the regulation of wounds (Yao et al., 2017), angiogenesis (Wang et al., 2017) and so on are shown in Supplementary Material 8. P311–TGF-β axis signaling was demonstrated to be important signaling that is related to the processes above. Moreover, mechanisms involving P311 in the expression of TGF-β were also found to be cell-specific. In NIH3T3 fibroblasts, P311 downregulated the expression of TGF-β1 and TGF-β2 binds to the LAP (latency associated protein) (Paliwal et al., 2004). In vascular smooth muscle cells, P311 binds eukaryotic translation initiation factor 3 subunit b (eIF3b) to promote the translation of the transforming growth factor β1–3 (TGF-β 1–3) (Badri et al., 2013; Yue et al., 2014). In EpSCs, P311 stimulated TGFβ1 expression by promoting TGFβ1 promoter methylation and by activating the TGFβ1 5′/3′UTR (Li et al., 2016). However, eIF6, an interacting protein of P311, downregulated the expression of TGFβ1 via H2A.Z occupancy and Sp1 recruitment in fibroblasts (Yang et al., 2015). Additionally, 13 predicted functions, in which P311 had never been implicated before, provided new insight into the function of P311 (Table 2).

### Predicted Functions Are Confirmed by Biological Experiments

With the proper biological experiments, we verified the predicted functions preliminarily. We confirmed the role of P311 in an inflammatory response, which was related to chemotaxis and immune responses (Table 2 and Supplementary Materials 9, 10), by checking the recruitment of inflammatory cells in the wound healing model. Compared to the P311 WT mice, the number of inflammatory cells was much lower in P311 KO mice, while the mRNA level of CD14 and CD16 was also much lower in P311 KO mice (Figure 3). It has been proven that P311 could regulate wound healing by accelerating reepithelization (Yao et al., 2017) or by affecting angiogenesis (Wang et al., 2017). It therefore implies that P311 might also regulate wound healing by affecting the inflammation response during wound healing.

The role of P311 in proliferation was identified by a proliferation assay and flow cytometry analysis of the cell cycle. The results showed that P311 promoted MPFs proliferation by enhancing cells into the S phase (Figure 4). The expression of P311 was also found in small-cell lung carcinoma (SCLC), large-cell neuroendocrine carcinoma (LCNEC) (Jones et al., 2004) and glioblastoma (Mariani et al., 2001), whose cells had a really high proliferation capability, which indirectly implied that P311 might regulate the proliferation of cells. This all indicated that P311 might regulate the proliferation of cells. However, further studies are required to validate and elucidate the underlying mechanisms.

Moreover, we also verified a role of P311 in coagulation. As we had observed the difference in the formation of scabs between P311 WT and P311 KO mice when we made the mice wound healing model (Wang et al., 2017), the bioinformatic analysis predicted the function of coagulation. These strongly implied a function of P311 in coagulation. We utilized Thromboelastography (TEG) to monitor the coagulation profile of blood after the burn injury. The result displayed that Angle, MA and G in the P311 KO-burn group were all higher than those in the P311 WT-burn group, which preliminarily verified that P311 might regulate coagulation. Further studies are needed to validate and elucidate the underlying mechanisms, which may suggest P311 as a potential therapeutic target for coagulation related diseases.

However, there were still several predicted functions, which were confirmed to not be associated with P311, through biological experiments. This indicated again that the bioinformatic analysis provides clues but does not validate it as truth. The strategy reported by our group can provide us with specific clues for follow-up biological experiments. Our study preliminarily found that P311 could be involved in inflammatory response, cell proliferation and coagulation. Further studies are required to validate and elucidate the underlying mechanism.

## Ethics Statement

All protocols involving animals were reviewed and approved by the Southwestern Hospital Institutional Review Board.

## Author Contributions

HL and GL made substantial contributions to the conception and design of the work. SW and XZ performed the majority of the experiments. RZ, YW, CS, and FH contributed to the collection, analysis and interpretation of the data for this study. SW wrote the first draft of the manuscript. YL, WH, and GL revised and edited the manuscript critically with important intellectual contributions. GL was responsible for obtaining funds. All authors read and approved the manuscript.

## Conflict of Interest Statement

The authors declare that the research was conducted in the absence of any commercial or financial relationships that could be construed as a potential conflict of interest. The reviewer LZ declared a shared affiliation, with no collaboration, with several of the authors, SW, XZ, FH, YL, RZ, YW, WH, HL, and GL, to the handling Editor at the time of review.
