# Corrigendum: Reconstruction and Functional Annotation of P311 Protein–Protein Interaction Network Reveals Its New Functions

**DOI:** 10.3389/fgene.2019.00818

**Published:** 2019-11-06

**Authors:** Song Wang, Xiaorong Zhang, Fen Hao, Yan Li, Chao Sun, Rixing Zhan, Ying Wang, Weifeng He, Haisheng Li, Gaoxing Luo

**Affiliations:** ^1^Institute of Burn Research, State Key Laboratory of Trauma, Burn and Combined Injury, Southwest Hospital, Third Military Medical University, Chongqing, China; ^2^Laboratory Center of Southwest Hospital, Third Military Medical University, Chongqing, China; ^3^The Sixth Resignation Cadre Sanatorium of Shandong Province Military Region, Qingdao, China; ^4^The 324th Hospital of Chinese People’s Liberation Army, Chongqing, China

**Keywords:** P311, protein–protein interaction networks, inflammatory response, cell proliferation, coagulation

There is an error in the ***Funding*** statement. The correct number for “China’s NSFC grants program” is “81630055.” A correction has therefore been made to the ***Funding*** statement and should read:

“This work was supported by grants from China’s NSFC grants program (81630055).”

Additionally, in the original article, the reference for “Becker et al., 2011” was incorrectly written as “Becker, E., Robisson, B., Chapple, C. E., Guénoche, A., and Brun, C. (2011). Multifunctional proteins revealed by overlapping clustering in protein interaction network. Bioinformatics 28, 84–90. doi: 10.1093/bioinformatics/btr621”. It should be “Becker, E., Robisson, B., Chapple, C. E., Guénoche, A., and Brun, C. (2012). Multifunctional proteins revealed by overlapping clustering in protein interaction network. Bioinformatics 28, 84–90. doi: 10.1093/bioinformatics/btr621”.

Lastly, the citation of Becker’s work was missing in the **Supplementary Material 4**. Corrections have been made by placing a copy of the code from Becker et al., Boinformatics, 2012 Jan 1;28(1):84–90, in the supplementary material along with an edited README file. Thus, corrections have also been made to the *Materials and Methods*, subsection *OCG (Overlapping Cluster Generator) Algorithm*, by removing the last sentence, which was redundant and to the first paragraph:

“The OCG algorithm was carried out by the software available in (Becker et al., 2012) (**Supplementary Material 4**).”

The authors apologize for these errors and state that they do not change the scientific conclusions of the article in any way. The original article has been updated.

