# Supplementary material for: Reconstruction and Functional Annotation of P311 Protein–Protein Interaction Network Reveals Its New Functions
Source: Front Genet. 2019 Feb 19;10:109. doi: 10.3389/fgene.2019.00109 (PMC6390203; doi:10.3389/fgene.2019.00109)
Supplement: Supplementary file 5 [file Data_Sheet_5.PDF]

# Dataset 1(80659 PPIs, 10229 proteins)

>Class 2 (73 nodes):

| | | | |
|---|---|---|---|
| ENSG00000000419 | ENSG00000005243 | ENSG00000005882 | ENSG00000058804 |
| ENSG00000064703 | ENSG00000067048 | ENSG00000067606 | ENSG00000079215 |
| ENSG00000082701 | ENSG00000092847 | ENSG00000095139 | ENSG00000095906 |
| ENSG00000100075 | ENSG00000100357 | ENSG00000100697 | ENSG00000101084 |
| ENSG00000102900 | ENSG00000103274 | ENSG00000103932 | ENSG00000104221 |
| ENSG00000109133 | ENSG00000110344 | ENSG00000113262 | ENSG00000118515 |
| ENSG00000123908 | ENSG00000124789 | ENSG00000125656 | ENSG00000127824 |
| ENSG00000128641 | ENSG00000131828 | ENSG00000132323 | ENSG00000134986 |
| ENSG00000136908 | ENSG00000136997 | ENSG00000140992 | ENSG00000142208 |
| ENSG00000143418 | ENSG00000144021 | ENSG00000144867 | ENSG00000145337 |
| ENSG00000149923 | ENSG00000150768 | ENSG00000152256 | ENSG00000155561 |
| ENSG00000156976 | ENSG00000160072 | ENSG00000163104 | ENSG00000163295 |
| ENSG00000163605 | ENSG00000165195 | ENSG00000166333 | ENSG00000166855 |
| ENSG00000174307 | ENSG00000175003 | ENSG00000179085 | ENSG00000179222 |
| ENSG00000179409 | ENSG00000181619 | ENSG00000181789 | ENSG00000182636 |
| ENSG00000182934 | ENSG00000184432 | ENSG00000184489 | ENSG00000186625 |
| ENSG00000187446 | ENSG00000196305 | ENSG00000196497 | ENSG00000196954 |
| ENSG00000197122 | ENSG00000197448 | ENSG00000204258 | ENSG00000204273 |
| ENSG00000258429 | | | |

>Class 69 (217 nodes):

| | | | |
|---|---|---|---|
| ENSG00000006075 | ENSG00000011465 | ENSG00000012963 | ENSG00000017427 |
| ENSG00000019991 | ENSG00000034053 | ENSG00000042781 | ENSG00000055955 |
| ENSG00000060656 | ENSG00000063660 | ENSG00000065135 | ENSG00000072062 |
| ENSG00000076716 | ENSG00000081052 | ENSG00000087245 | ENSG00000088179 |
| ENSG00000088926 | ENSG00000092009 | ENSG00000099985 | ENSG00000100299 |
| ENSG00000100665 | ENSG00000100985 | ENSG00000101294 | ENSG00000101425 |
| ENSG00000102265 | ENSG00000102312 | ENSG00000102962 | ENSG00000102970 |
| ENSG00000102996 | ENSG00000103811 | ENSG00000104368 | ENSG00000105143 |
| ENSG00000105664 | ENSG00000107140 | ENSG00000108688 | ENSG00000108700 |
| ENSG00000108702 | ENSG00000109758 | ENSG00000109861 | ENSG00000113889 |
| ENSG00000113905 | ENSG00000114353 | ENSG00000115414 | ENSG00000115596 |
| ENSG00000115884 | ENSG00000116014 | ENSG00000116157 | ENSG00000117984 |
| ENSG00000121552 | ENSG00000121807 | ENSG00000122194 | ENSG00000122852 |
| ENSG00000122854 | ENSG00000122862 | ENSG00000123384 | ENSG00000123572 |
| ENSG00000124334 | ENSG00000125084 | ENSG00000125810 | ENSG00000125966 |
| ENSG00000126353 | ENSG00000126562 | ENSG00000127955 | ENSG00000128218 |
| ENSG00000128242 | ENSG00000129048 | ENSG00000129277 | ENSG00000129518 |

| | | | |
|---|---|---|---|
| ENSG00000130656 | ENSG00000131187 | ENSG00000132671 | ENSG00000132692 |
| ENSG00000134352 | ENSG00000134871 | ENSG00000134986 | ENSG00000135047 |
| ENSG00000135919 | ENSG00000136770 | ENSG00000137077 | ENSG00000137486 |
| ENSG00000137673 | ENSG00000137674 | ENSG00000137745 | ENSG00000137801 |
| ENSG00000138207 | ENSG00000138448 | ENSG00000139219 | ENSG00000139874 |
| ENSG00000140092 | ENSG00000140443 | ENSG00000141756 | ENSG00000142192 |
| ENSG00000143387 | ENSG00000144366 | ENSG00000144648 | ENSG00000145423 |
| ENSG00000145623 | ENSG00000145681 | ENSG00000146648 | ENSG00000146674 |
| ENSG00000147168 | ENSG00000148346 | ENSG00000148702 | ENSG00000149968 |
| ENSG00000152377 | ENSG00000153944 | ENSG00000156103 | ENSG00000156234 |
| ENSG00000157005 | ENSG00000157227 | ENSG00000157766 | ENSG00000158859 |
| ENSG00000159189 | ENSG00000159403 | ENSG00000159788 | ENSG00000160213 |
| ENSG00000160294 | ENSG00000160791 | ENSG00000160803 | ENSG00000160862 |
| ENSG00000161570 | ENSG00000161573 | ENSG00000161574 | ENSG00000162009 |
| ENSG00000162337 | ENSG00000162434 | ENSG00000162552 | ENSG00000162604 |
| ENSG00000162706 | ENSG00000163347 | ENSG00000163386 | ENSG00000163453 |
| ENSG00000163520 | ENSG00000163536 | ENSG00000163631 | ENSG00000163661 |
| ENSG00000163737 | ENSG00000163823 | ENSG00000164344 | ENSG00000164733 |
| ENSG00000165215 | ENSG00000165219 | ENSG00000166634 | ENSG00000166670 |
| ENSG00000166913 | ENSG00000167236 | ENSG00000167346 | ENSG00000167711 |
| ENSG00000167748 | ENSG00000167751 | ENSG00000167880 | ENSG00000168878 |
| ENSG00000169031 | ENSG00000169245 | ENSG00000169429 | ENSG00000169575 |
| ENSG00000170027 | ENSG00000170498 | ENSG00000171033 | ENSG00000171560 |
| ENSG00000171603 | ENSG00000172724 | ENSG00000172757 | ENSG00000173011 |
| ENSG00000173020 | ENSG00000173369 | ENSG00000173372 | ENSG00000173432 |
| ENSG00000173531 | ENSG00000173636 | ENSG00000175279 | ENSG00000177283 |
| ENSG00000177479 | ENSG00000179934 | ENSG00000180210 | ENSG00000180871 |
| ENSG00000181856 | ENSG00000182314 | ENSG00000182326 | ENSG00000182871 |
| ENSG00000183473 | ENSG00000183625 | ENSG00000183695 | ENSG00000183813 |
| ENSG00000184451 | ENSG00000184937 | ENSG00000185245 | ENSG00000185303 |
| ENSG00000185918 | ENSG00000186676 | ENSG00000186965 | ENSG00000187049 |
| ENSG00000187122 | ENSG00000187498 | ENSG00000187678 | ENSG00000187908 |
| ENSG00000188153 | ENSG00000188170 | ENSG00000188488 | ENSG00000196136 |
| ENSG00000196535 | ENSG00000196611 | ENSG00000197249 | ENSG00000197561 |
| ENSG00000197565 | ENSG00000197635 | ENSG00000197641 | ENSG00000197711 |
| ENSG00000198670 | ENSG00000204982 | ENSG00000205021 | ENSG00000205595 |
| ENSG00000206047 | ENSG00000206073 | ENSG00000206403 | ENSG00000211899 |
| ENSG00000242372 | | | |

>Class 430 (55 nodes):

| | | | |
|---|---|---|---|
| ENSG00000014138 | ENSG00000073111 | ENSG00000076003 | ENSG00000085840 |
| ENSG00000087263 | ENSG00000088298 | ENSG00000095319 | ENSG00000100297 |
| ENSG00000101158 | ENSG00000101868 | ENSG00000104738 | ENSG00000106399 |
| ENSG00000111229 | ENSG00000112118 | ENSG00000115942 | ENSG00000115947 |

ENSG00000117748 ENSG00000123374 ENSG00000126088 ENSG00000126267
ENSG00000127311 ENSG00000130429 ENSG00000132383 ENSG00000132744
ENSG00000133627 ENSG00000134086 ENSG00000134986 ENSG00000136810
ENSG00000137996 ENSG00000138071 ENSG00000139687 ENSG00000140859
ENSG00000140968 ENSG00000145425 ENSG00000147383 ENSG00000156983
ENSG00000158710 ENSG00000162704 ENSG00000163466 ENSG00000163479
ENSG00000164815 ENSG00000166508 ENSG00000167325 ENSG00000168310
ENSG00000170604 ENSG00000171848 ENSG00000171877 ENSG00000173068
ENSG00000173221 ENSG00000175104 ENSG00000182934 ENSG00000184432
ENSG00000184489 ENSG00000197442 ENSG00000204086

>Class 454 (31 nodes):
ENSG00000010810 ENSG00000039068 ENSG00000064012 ENSG00000075711
ENSG00000080815 ENSG00000092445 ENSG00000092820 ENSG00000095321
ENSG00000096433 ENSG00000107282 ENSG00000120498 ENSG00000124181
ENSG00000127831 ENSG00000132535 ENSG00000133250 ENSG00000134986
ENSG00000137672 ENSG00000138741 ENSG00000145675 ENSG00000147246
ENSG00000148943 ENSG00000152990 ENSG00000164305 ENSG00000168036
ENSG00000173992 ENSG00000178922 ENSG00000182132 ENSG00000184408
ENSG00000197122 ENSG00000197471 ENSG00000197892

# Dataset 2(110707 PPIs, 9606 proteins)

>Class 590 (31 nodes):
ENSP00000221700 ENSP00000222214 ENSP00000222982 ENSP00000223023
ENSP00000224356 ENSP00000225655 ENSP00000225740 ENSP00000226230
ENSP00000229922 ENSP00000231887 ENSP00000232461 ENSP00000238561
ENSP00000242592 ENSP00000242729 ENSP00000244043 ENSP00000255082
ENSP00000280886 ENSP00000282516 ENSP00000291598 ENSP00000292896
ENSP00000294635 ENSP00000301180 ENSP00000314901 ENSP00000315757
ENSP00000328023 ENSP00000339787 ENSP00000350012 ENSP00000353791
ENSP00000358765 ENSP00000378996 ENSP00000400184

>Class 666 (19 nodes):
ENSP00000206542 ENSP00000211998 ENSP00000215832 ENSP00000216911
ENSP00000220003 ENSP00000220764 ENSP00000221233 ENSP00000226091
ENSP00000230990 ENSP00000239891 ENSP00000245185 ENSP00000248673
ENSP00000274376 ENSP00000309845 ENSP00000323178 ENSP00000334061
ENSP00000334840 ENSP00000378996 ENSP00000415774

>Class 1004 (20 nodes):

ENSP00000209728 ENSP00000218032 ENSP00000223324 ENSP00000225792
ENSP00000228872 ENSP00000232003 ENSP00000236957 ENSP00000238146
ENSP00000241041 ENSP00000243673 ENSP00000256433 ENSP00000261537
ENSP00000318227 ENSP00000332931 ENSP00000350263 ENSP00000361502
ENSP00000378288 ENSP00000378996 ENSP00000386292 ENSP00000443772

>Class 1215  (14 nodes):
ENSP00000216442 ENSP00000222286 ENSP00000229402 ENSP00000233084
ENSP00000233545 ENSP00000234396 ENSP00000244007 ENSP00000265723
ENSP00000283426 ENSP00000363559 ENSP00000376544 ENSP00000377401
ENSP00000378996 ENSP00000417378
