# Supplementary figures and images for: Reconstruction and Functional Annotation of P311 Protein–Protein Interaction Network Reveals Its New Functions
Source: Front Genet. 2019 Feb 19;10:109. doi: 10.3389/fgene.2019.00109 (PMC6390203; doi:10.3389/fgene.2019.00109)

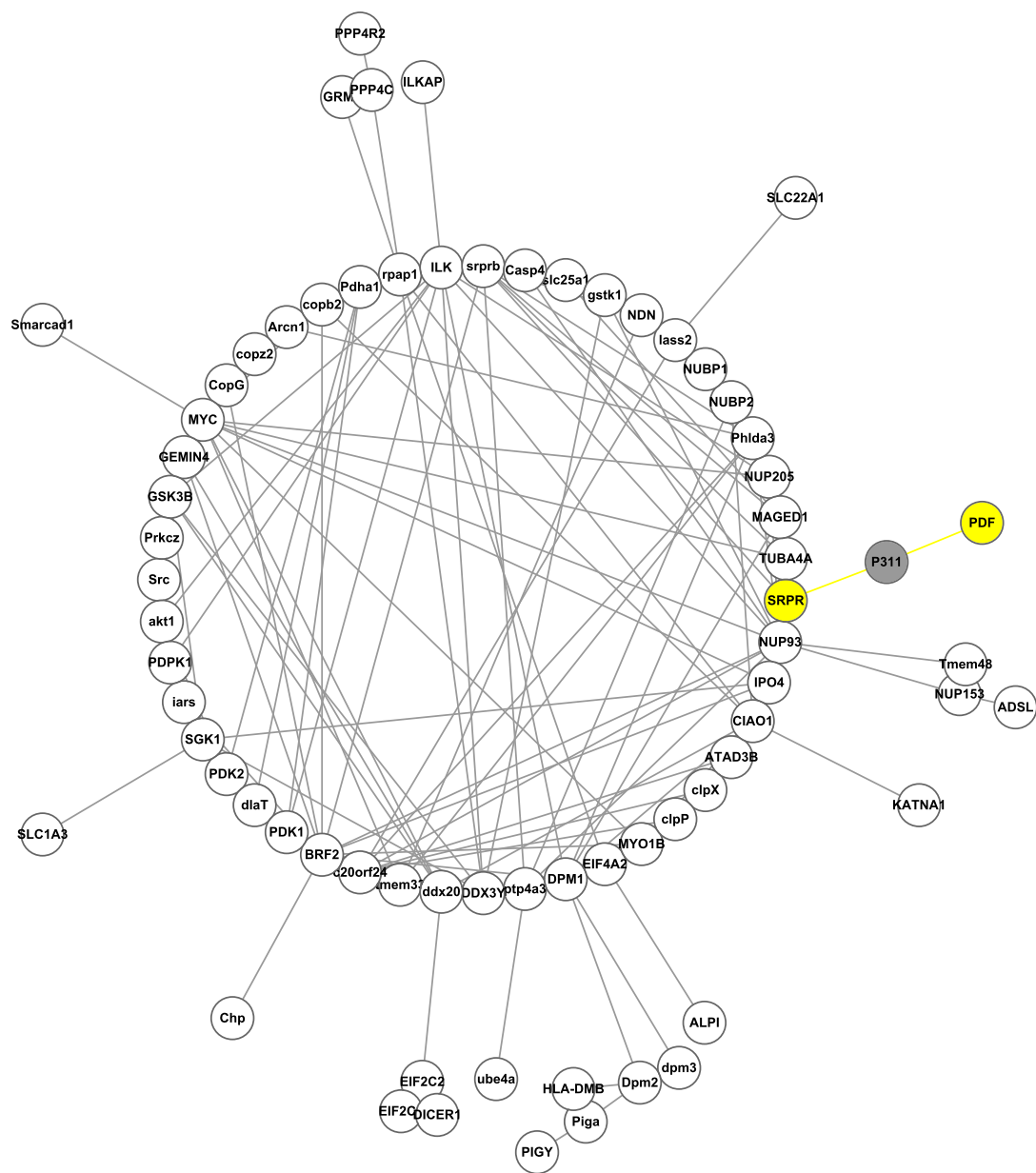

M1

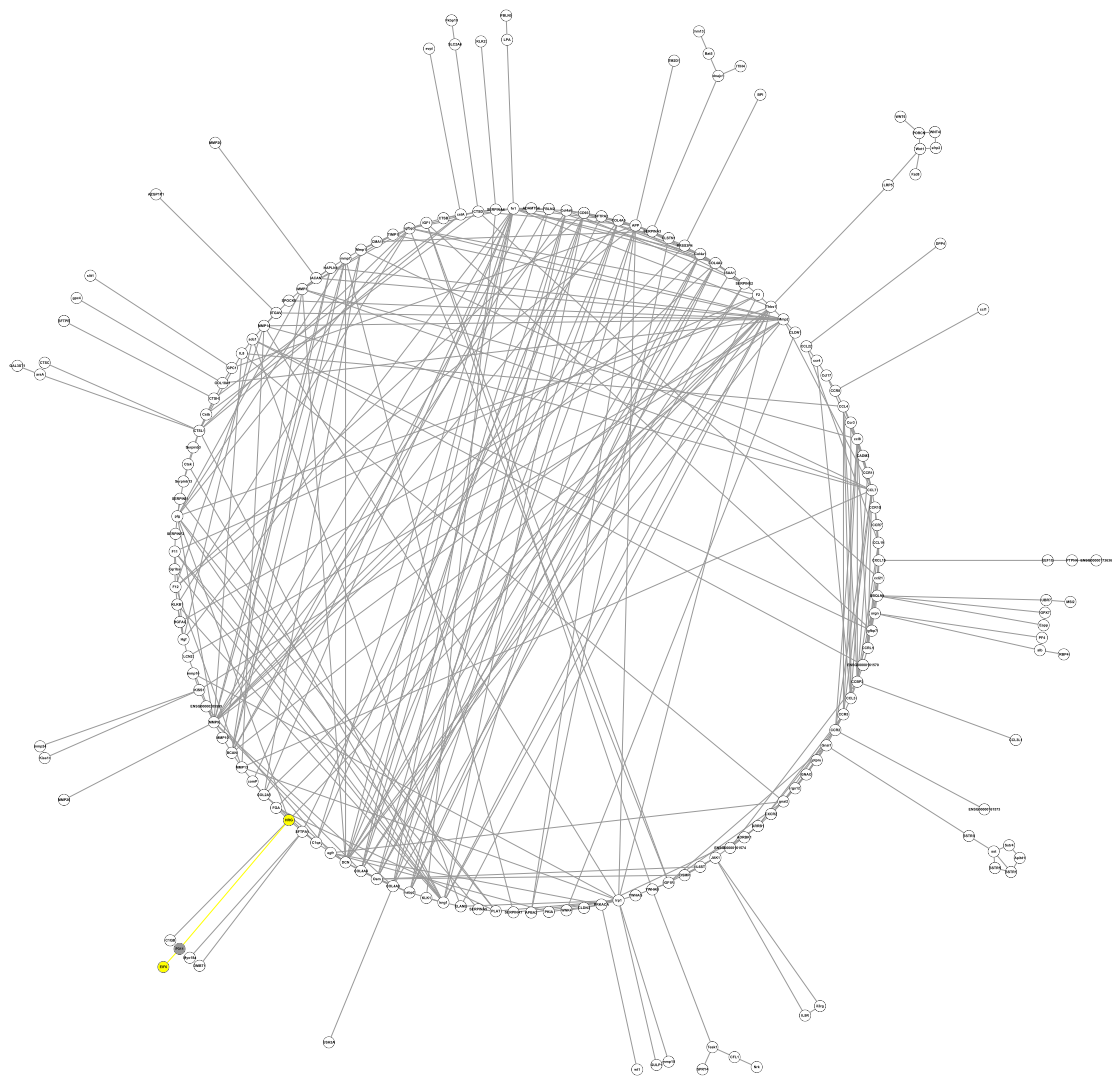

**M2**

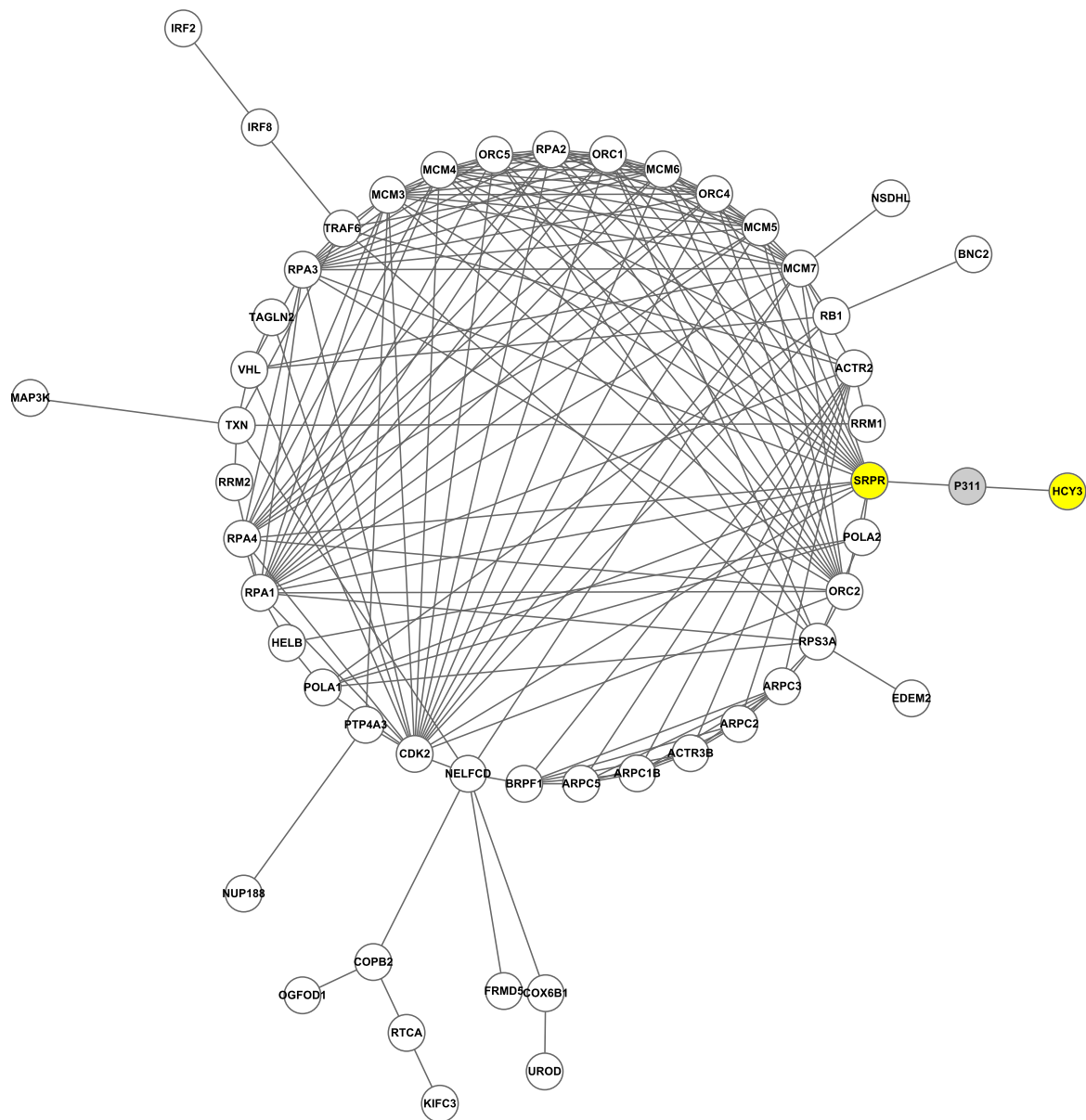

**M3**

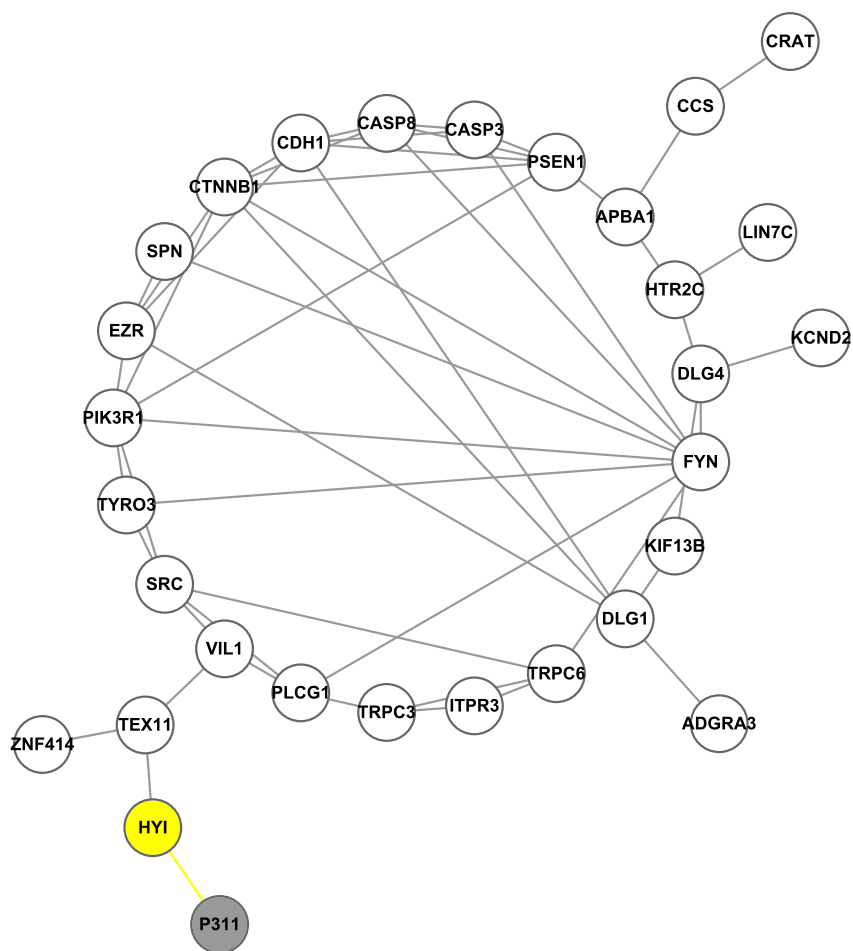

**M4**

Supplement: Supplementary file 6 [file Data_Sheet_6.PDF]

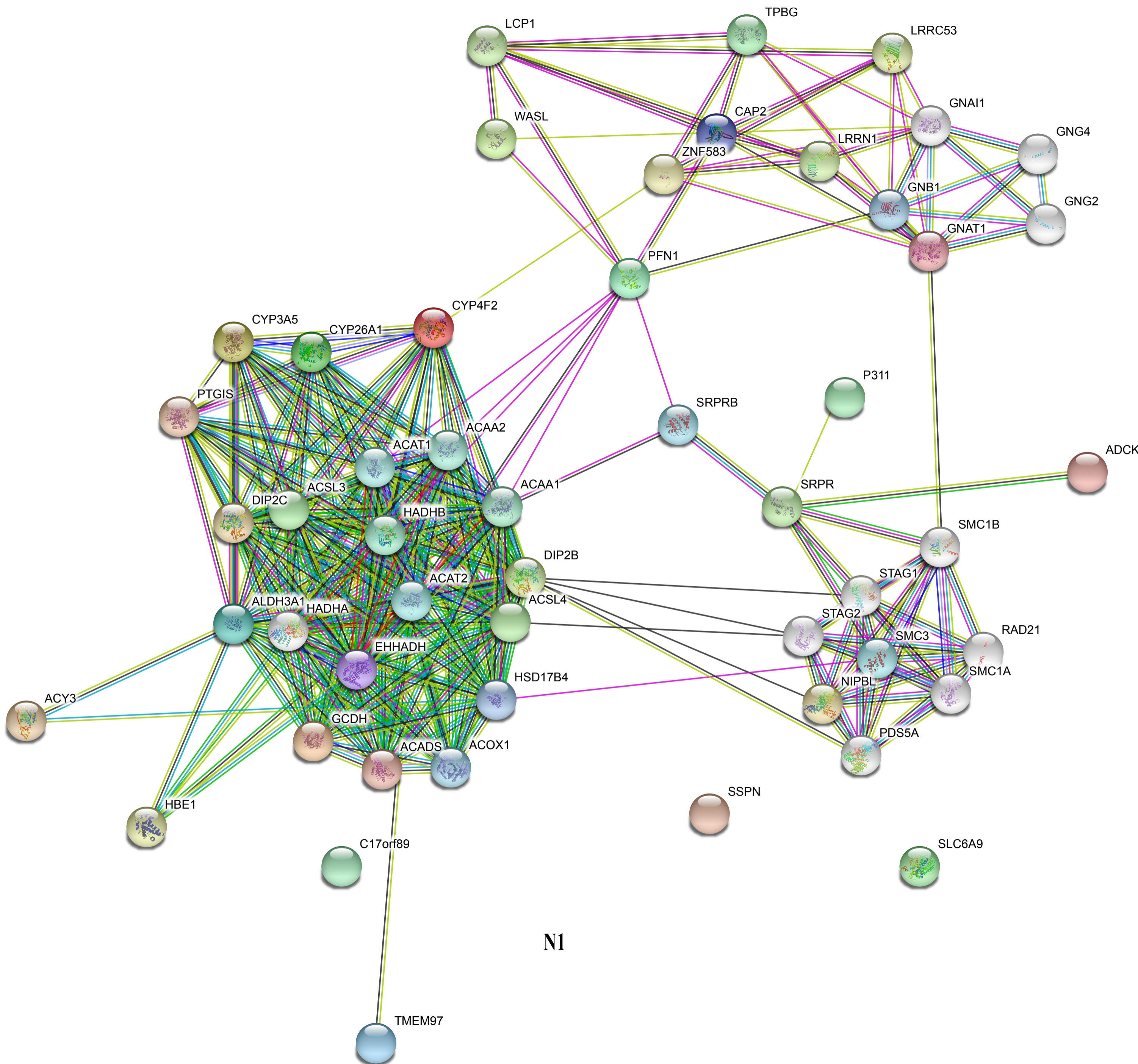

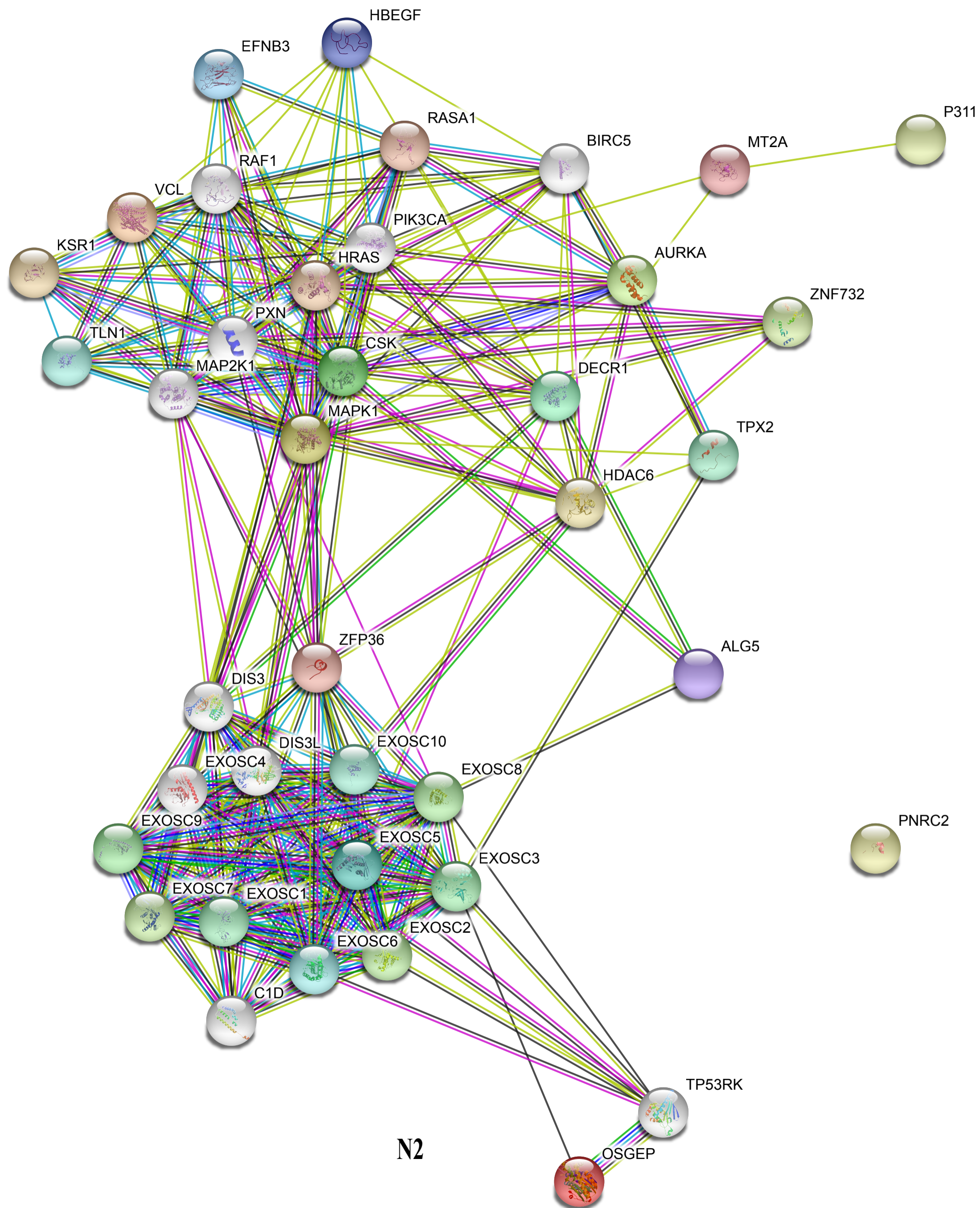

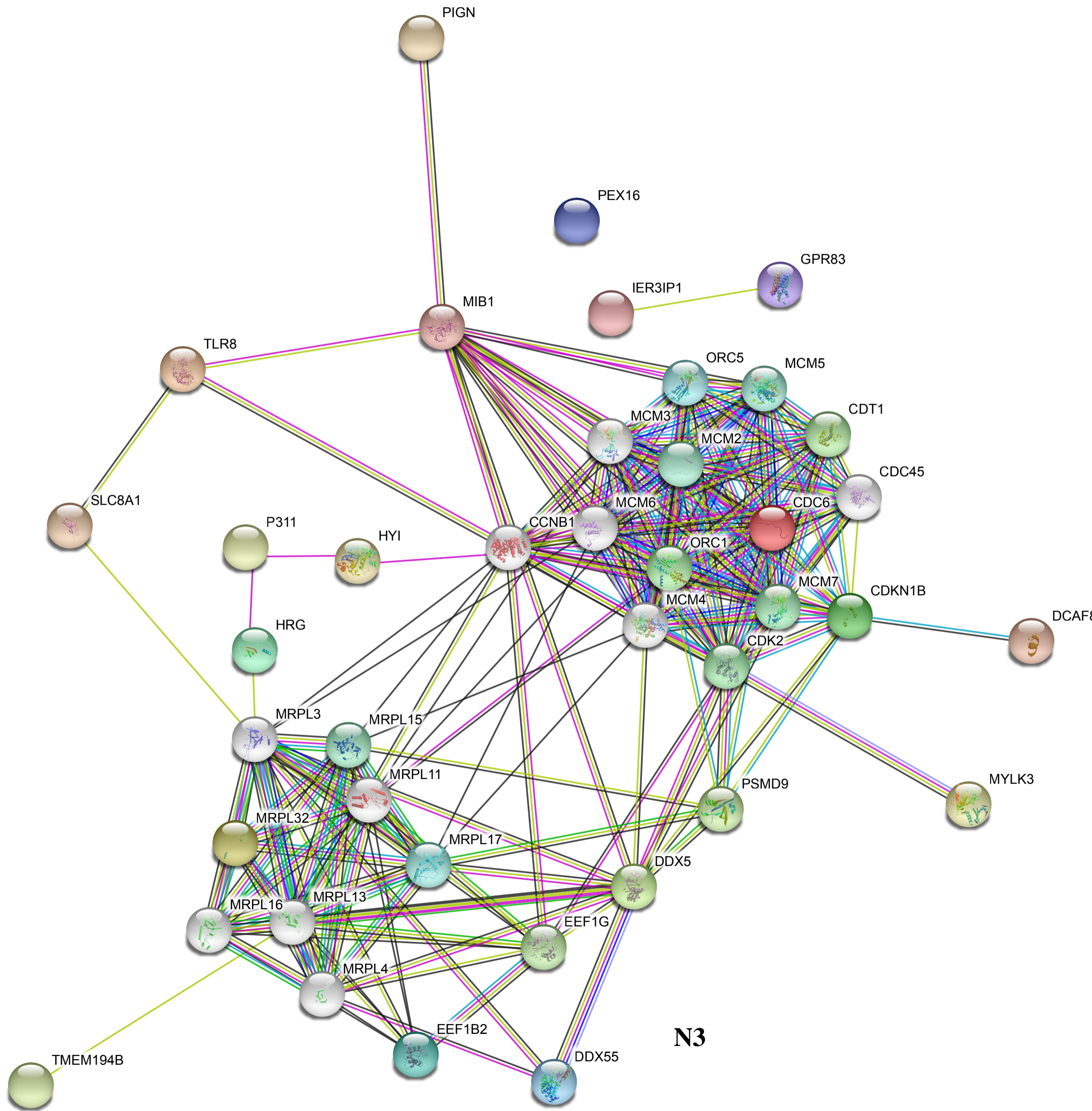

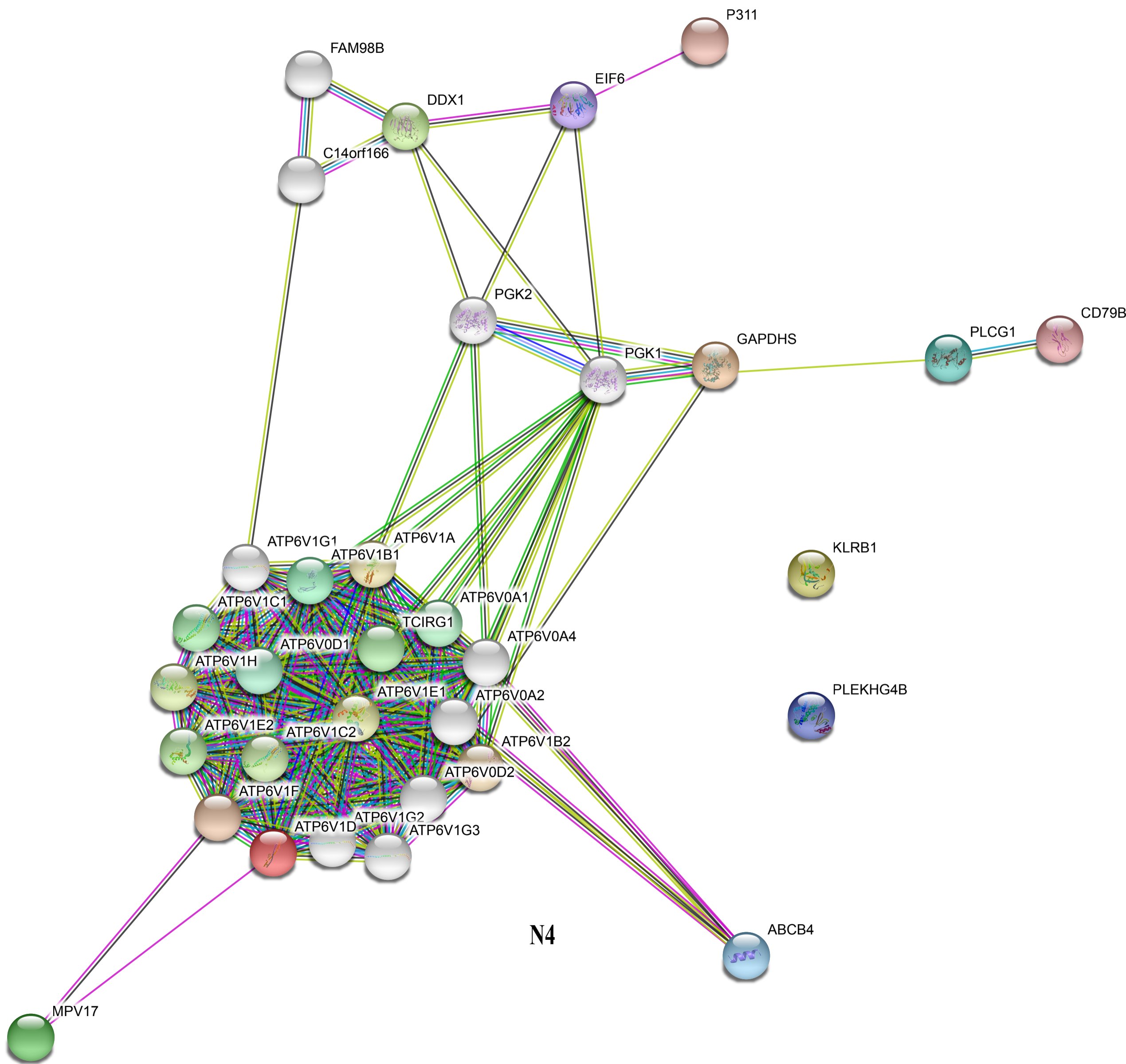

Supplement: Supplementary file 7 [file Data_Sheet_7.PDF]
