# Supplementary material for: Reconstruction and Functional Annotation of P311 Protein–Protein Interaction Network Reveals Its New Functions
Source: Front Genet. 2019 Feb 19;10:109. doi: 10.3389/fgene.2019.00109 (PMC6390203; doi:10.3389/fgene.2019.00109)
Supplement: Supplementary file 8 [file Data_Sheet_8.PDF]

Known function of P311
**Regulation of cell migration**[1, 2].
**Reponds to wounding** [1]**,angiogenesis**[3].
**Regulation of nervous system development**[4]**, regeneration**[5]**.**
**Regulation of blood pressure homeostasis**[6].
**Actin cytoskeleton organization**[2] **and muscle constraction**[6].
**Regulation of collagen catabolic process**[7].
**Regulation of translation** [7, 8].
**Regulation of development of fibrosis**[9, 10]**.**
**Regulation of cancer progression**[11]**.**

References:

[1]. Yao, Z., et al., P311 Accelerates Skin Wound Reepithelialization by Promoting Epidermal Stem Cell Migration Through RhoA and Rac1 Activation. Stem Cells Dev, 2017. 26(6): p. 451-460.

[2]. McDonough, W.S., N.L. Tran and M.E. Berens, Regulation of glioma cell migration by serine-phosphorylated P311. Neoplasia, 2005. 7(9): p. 862-72.

[3]. Wang, S., et al., P311 Deficiency Leads to Attenuated Angiogenesis in Cutaneous Wound Healing. Front Physiol, 2017. 8: p. 1004.

[4]. Fujitani, M., et al., P311 accelerates nerve regeneration of the axotomized facial nerve. J Neurochem, 2004. 91(3): p. 737-44.

[5]. Zhao, L., et al., Identification of P311 as a potential gene regulating alveolar generation. Am J Respir Cell Mol Biol, 2006. 35(1): p. 48-54.

[6]. Badri, K.R., et al., Blood pressure homeostasis is maintained by a P311-TGF-beta axis. J Clin Invest, 2013. 123(10): p. 4502-12.

[7]. Cheng, T., et al., Neuronal Protein 3.1 Deficiency Leads to Reduced Cutaneous Scar Collagen Deposition and Tensile Strength due to Impaired Transforming Growth Factor-beta1 to -beta3 Translation. Am J Pathol, 2017. 187(2): p. 292-303.

[8]. Li, H., et al., P311 induces the transdifferentiation of epidermal stem cells to myofibroblast-like cells by stimulating transforming growth factor beta1 expression. Stem Cell Res Ther, 2016. 7(1): p. 175.

[9]. Yao, Z., et al., P311 promotes renal fibrosis via TGFbeta1/Smad signaling. Sci Rep, 2015. 5: p. 17032.

[10]. Wang, F., et al., Expression of P311, a transforming growth factor beta latency-associated protein-binding protein, in human kidneys with IgA nephropathy. Int Urol Nephrol, 2010. 42(3): p. 811-9.
[11]. McDonough, W.S., N.L. Tran and M.E. Berens, Regulation of glioma cell migration by serine-phosphorylated P311. Neoplasia, 2005. 7(9): p. 862-72.
